# Pure Air

**Published:** 1898-02

**Authors:** Geo. J. Monroe

**Affiliations:** Louisville, Ky.


### Pure Air.

BY GEO. J. MONROE, M.D., LOUISVILLE, KY.

In house, shop, office, store, schools, churches, halls or any place where the human family lives or congregates, we should see to it that the air is pure. Pure air is essential that we may live, and the purer the air the more joy we will get out of life and the longer we will live. If a manufacturing establishment where thousands congregate and remain for hours is poorly ventilated, or if pure air does not enter it freely, we will have as a result thousands in poor health. If our school-rooms are permeated with vitiated air we have sickly school children. If our stores and other places of business have not good, fresh, wholesome air we have poor and unfortunate business men and clerks. Men or women who are obliged to live in a poisonous atmosphere are irritable, cross and disagreeable, nearly always dyspeptic, and are more apt to be failures than their next-door neighbor, who, perchance, has not the natural business qualities they have; they breathe a pure air, however, hence their brains are clear, active and under complete control. Families who live in poorly-ventilated houses are always cross and snarly. They are constantly quarreling amongst themselves, and always complaining of some physical ailment. Headache is probably one of the most frequent accompaniments of living in impure air. Digestion is usually poor, and constipation generally exists. Any one living in a poisonous atmosphere is devoid of energy. They desire to sleep, or at any rate to remain quiet the most of the time. Their sleep is broken; they are tormented with horrible dreams. When they finally awaken in the morning they are tired and worn out. They have a disagreeable taste in their mouths, tongue furred, and almost always a frontal headache and some backache. The skin is dark and lifeless, the eyes surrounded by a dark ring. The eye-sight very soon becomes affected. An oppressed, depressed feeling predominates throughout the entire system. I believe that impure air will produce a sluggish condition of the liver; in fact, we seldom ever have poor digestion and an active liver at the same time. The one sympathizes with the other. Poor digestion causes a sluggish liver, and, *per contra*, a sluggish liver causes poor digestion. Neither do we have, as a rule, a diseased stomach and liver without having more or less trouble with the kidneys. This kidney trouble may be simply sympathetic, or we may have organic disease. We should see to it that all public as well as private buildings should be well ventilated.

In order to have pure air enter buildings it is necessary to have pure air outside; certainly, if the air is contaminated with filth, decaying animal or vegetable matter, or poor sewerage, manure and other nuisances, we have difficulty in obtaining pure air, and the result is disease. This being the case outside, we need not expect pure air inside.

The immense amount of smoke which we have in every city from the combustion of soft coal evidently is a disease-producing element. How often we notice sore throats, coughs and other nasal and bronchial troubles so soon as we light our fires in the fall or early winter, when we begin to burn soft coal, and natural gas is much worse. I am not certain that coal smoke shortens life very much, but I am quite certain it makes many a one a partial invalid during the winter months. I have not failed in twenty years having a cough begin every fall when soft coal fires were started and continue all winter. I was told by the Nestor of medicine, N. S. Davis, of Chicago, over twenty years ago, that I had better arrange my business for my departure from this earth, as I would not live a year. I am still living, and I do not think this winter's cough is going to shorten my life very much, yet I must admit it is very annoying. I attributed this cough formerly to the effect of cold weather, but I am now satisfied it is occasioned from soot and the smoke of soft coal.

Any one can easily prove that the air is saturated with coal soot and smoke. Let him clean his nose thoroughly before leaving home. Let him walk a mile to his place of business and again clean his nose, and he will be surprised at the amount of soot that has collected upon the mucous membrane. This foreign substance can not be conducive to health. Some enterprising and inventive genius, it seems to me, ought to be able to invent some method whereby all this carbon with which the air is loaded could be burned or destroyed. I understand that some such invention has been made and is in use in some of our large cities. If it is a success it ought to be adopted in every city. I doubt if it as yet has been a complete success.

School-houses and churches should especially be well ventilated. The people are seeing the necessity of this, especially in schools. There is not yet much attention given to churches and theaters. How common it is to go to church and find a musty, sickening odor! The atmosphere is saturated by all or nearly all of the carbonic acid gas which has been exhaled the Sunday previous. During the week the church has been hermetically sealed; no air has been allowed to enter. With this amount of carbonic acid gas no wonder the congregation has been inattentive and sleepy. If the weather is cold a great fire is built, burning up the little oxygen that still remains in the room. This fire also stirs up the carbonic acid gas which has accumulated for some time previous. No wonder that the ministers become exhausted in delivering their Sunday sermons, and no wonder that the congregation is sleepy and listless. Ask many a one who has attended church on Sunday what the sermon was about, and they can not possibly tell you. This is simply on account of the impure air in the church acting as a narcotic to the brain. No one can be a good, industrious and attentive Christian and attend church where the air is impure.

We keep our houses in the winter time as tightly closed as possible in order to retain the artificial heat of our stoves, grates and furnaces. We do not take into consideration the vitiated air in which we are living, which is slowly but surely destroying our lives, destroying our energy and ambition, and materially affecting our peace of mind, making us discontented and unhappy. Many often wonder why they feel so badly in the morning when first awakening, or on getting up; "tired" does not express the feeling. If they would reflect a moment they would remember that every avenue where outside air might enter the sleeping-room is hermetically sealed. The windows are never opened, or at any rate at night. The occupants of the room have been breathing and re-breathing the atmosphere of the room until all of the oxygen of the air has been exhausted. They are simply trying to breathe carbonic acid gas. This gas, it is well known, poisons the blood. It has a tendency to produce a sleepy condition. If there is much of it it will not sustain life. No wonder a person is tired who has for any length of time breathed this gas. Many, from an excess of this gas, pass into that sleep which knows no wakening. Witness the great number who died from it in the "Black Hole" of Calcutta. So many were confined in this one room, which only had one small window for the entrance of air, that the air was soon contaminated with carbonic acid gas from their own lungs that there was not sufficient oxygen to continue life. Only a few out of the hundred and forty remained alive.

Carbonic acid gas may be further illustrated by its accumulation in deep wells and in cellars and mines. If there is not sufficient oxygen to sustain life we can have no combustion. If a candle will not burn the genus homo had better keep out of this gas. If it will put out a candle it will put out life, for where a candle will not burn life can not exist. Carbonic acid gas becomes sometimes so heavy that you can dip it, as well as you can water. This experiment may be proved by dipping a pail of it from a deep well where it has been accumulating for any length of time. Place a dozen lighted candles in a trough at an angle of forty-five degrees. Turn your carbonic gas in at the upper end of the trough and you will find every light will be extinguished. This, of course, is proof positive that life can not continue in an atmosphere saturated with carbonic acid gas.

I do not think it possible for anybody to have too much pure air. We should endeavor to have this pure air by all means in our power. Give us pure air and pure water and we can withstand impurities of other kinds with a greater degree of strength than we can if the air is contaminated and the water polluted, which we very often have to drink. Good Lord, deliver us from the many impurities we have to contend with.

